# Towards improved health service quality in Tanzania: contribution of a supportive supervision approach to increased quality of primary healthcare

**DOI:** 10.1186/s12913-019-4648-2

**Published:** 2019-11-20

**Authors:** Sabine Renggli, Iddy Mayumana, Dominick Mboya, Christopher Charles, Christopher Mshana, Flora Kessy, Tracy R. Glass, Christian Lengeler, Alexander Schulze, Ann Aerts, Constanze Pfeiffer

**Affiliations:** 1 Department of Epidemiology and Public Health, Swiss Tropical and Public Health Institute, P.O. Box, 4002, Basel, Switzerland; 2 University of Basel, Basel, Switzerland; 3 Ifakara Health Institute, Dar es Salaam/Ifakara, United Republic of Tanzania; 4 Swiss Agency for Development and Cooperation, Berne, Switzerland; 5 Novartis Foundation, Basel, Switzerland

**Keywords:** Quality of care, Quality improvement approach, Tanzania, Electronic tool, Supportive supervision, Universal health coverage

## Abstract

**Background:**

Universal Health Coverage only leads to the desired health outcomes if quality of health services is ensured. In Tanzania, quality has been a major concern for many years, including the problem of ineffective and inadequate routine supportive supervision of healthcare providers by council health management teams. To address this, we developed and assessed an approach to improve quality of primary healthcare through enhanced routine supportive supervision.

**Methods:**

Mixed methods were used, combining trends of quantitative quality of care measurements with qualitative data mainly collected through in-depth interviews. The former allowed for identification of drivers of quality improvements and the latter investigated the perceived contribution of the new supportive supervision approach to these improvements.

**Results:**

The results showed that the new approach managed to address quality issues that could be solved either solely by the healthcare provider, or in collaboration with the council. The new approach was able to improve and maintain crucial primary healthcare quality standards across different health facility level and owner categories in various contexts.

**Conclusion:**

Together with other findings reported in companion papers, we could show that the new supportive supervision approach not only served to assess quality of primary healthcare, but also to improve and maintain crucial primary healthcare quality standards. The new approach therefore presents a powerful tool to support, guide and drive quality improvement measures within council. It can thus be considered a suitable option to make routine supportive supervision more effective and adequate.

## Background

Since the publication of the World Health Report in 2010 there is growing ambition in many countries for progress towards Universal Health Coverage (UHC) [1, 2]. This was further stimulated through the formulation of UHC as one of the prominent targets of the health-related Sustainable Development Goal 3 [3]. However, there is no benefit to UHC if poor quality of care leads to unwillingness of people to use services [4]. And even if services are accessed and used, studies suggest that poor quality is undermining health outcomes [5–8]. Consequently, health services need to be of sufficient quality to achieve the desired outcomes and therefore improving quality must be of highest priority [4, 9, 10]. One of the main challenges resulting in weak quality in low- and middle-income countries is the lack of enough, well-trained and motivated staff with adequate financial and physical resources to provide basic health services [11, 12]. Another problem is insufficient resources and/or ineffective and inefficient allocation of limited resources [12, 13]. Additionally, upon quality assessments district managers and healthcare providers seldom receive feedback on the performance of their facilities. As a result, assessment results are rarely translated into appropriate quality improvement measures [14]. It was moreover reported that many assessments seemed to measure donor funded programs rather than country owned initiatives, leading to parallel monitoring structures that burden the system [14, 15]. In Tanzania, given the expansion of health services, quality of care has become a major concern for many years [16]. Some of the issues are low standards of hygiene and sanitation, insufficient health infrastructure, poor healthcare waste disposal, low motivation of health workers, inadequate adherence to professional and ethical conduct, as well as a know-do gap amongst health workers [16, 17]. The last point refers to the gap between what health workers know and what they actually do [18]. Missing ownership of quality improvement measures at facility level and poor feedback on quality developments at council level are further issues found in Tanzania [16, 17]. Also, Council Health Management Teams (CHMTs), who are in charge of managing services provided within their council, are often conducting routine supportive supervision of healthcare providers inadequately and ineffectively [16, 17, 19]. Amongst other things, the main problems of routine CHMT supportive supervision are infrequency, fragmentation, incompleteness and inconsistency as well as a focus on quantity (reviewing record books) instead of quality (service delivery processes) [16, 20–27]. Supportive supervision was shown to promote quality improvements in several low resource settings, but strongly depends on the way it is conducted [28–38]. Already in the Tanzanian Health Sector Strategic Plan III (HSSP) (2009–2015) the need to put quality improvement systems in place was stipulated [39]. The topic received even greater attention in the subsequent HSSP IV (2015–2020) [40]. According to this plan, operationalization of quality improvement ought to be done through the introduction of a performance-based certification system, clients’ charters, pay-for-performance (P4P) schemes and an integrated quality improvement program. The latter is supposed to include a national quality improvement toolkit and monitoring system, facility self-assessments and comprehensive supportive supervision, mentoring and coaching [40]. The plan is backed-up by a series of basic standards for health facilities at each level of the Tanzanian healthcare system [41–44]. The HSSP IV as well specifies the need for harmonizing, coordinating and integrating the improvement initiatives of the disease specific national control programs [40]. Apart from these initiatives, there are also rather uncoordinated and sometimes duplicative quality improvement approaches from other stakeholders [16, 17, 45]. These approaches rely usually on external assessments conducted in the frame of certification or accreditation procedures, on trainings with subsequent follow-up visits to health facilities or on self-assessments done at health facilities [13, 16, 25, 46–55]. To the best of our knowledge, none of the documented approaches looked at routine CHMT supportive supervision. Thus, given the need to improve quality of care and strengthen routine supportive supervision of healthcare providers through their CHMT, we systematically evaluated a new supportive supervision approach that aimed to serve this purpose.

## Methods

### Study setting

**Fig. 1:**
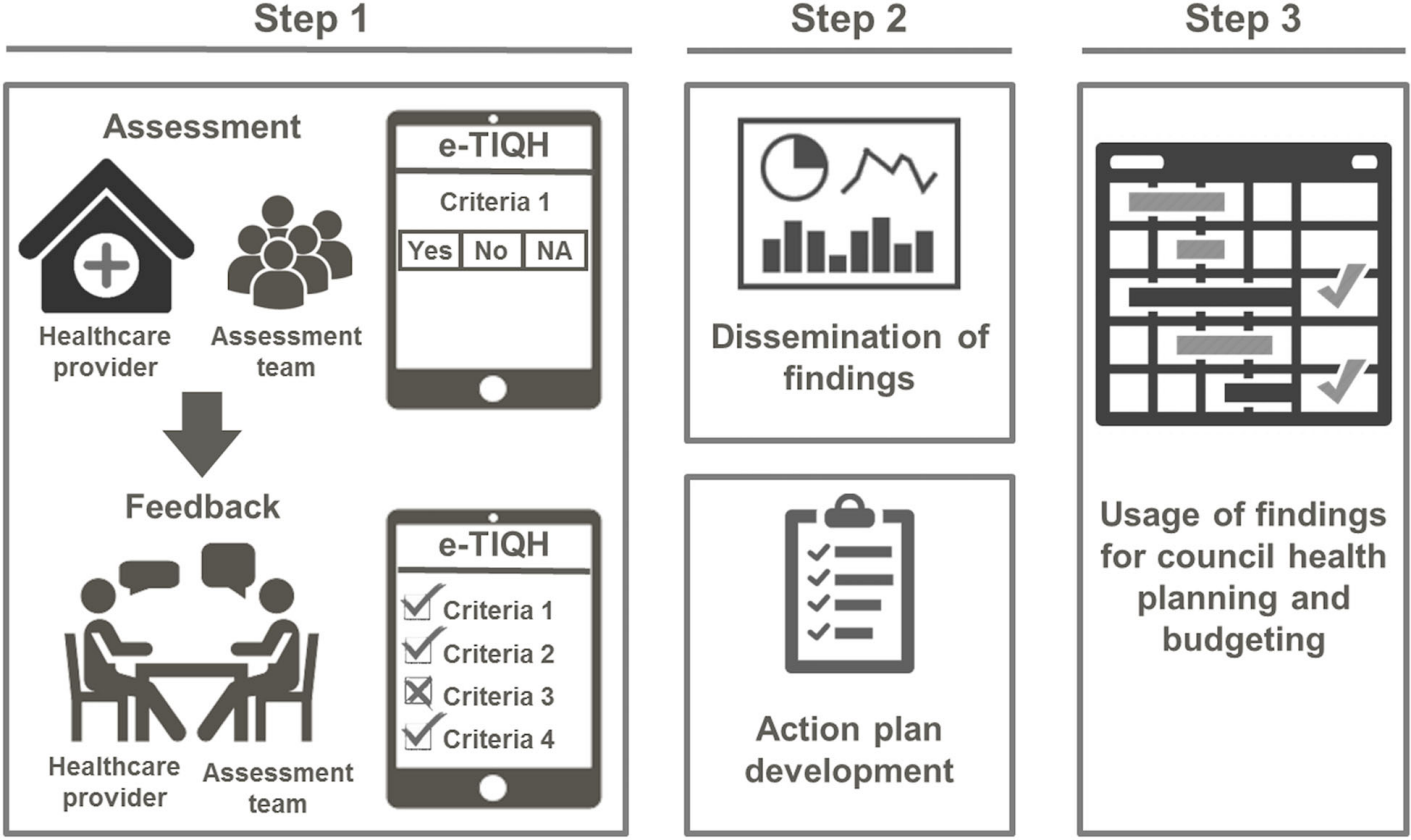
Chart of the three-stage process of the e-TIQH supportive supervision approach [57]

**Fig. 2:**
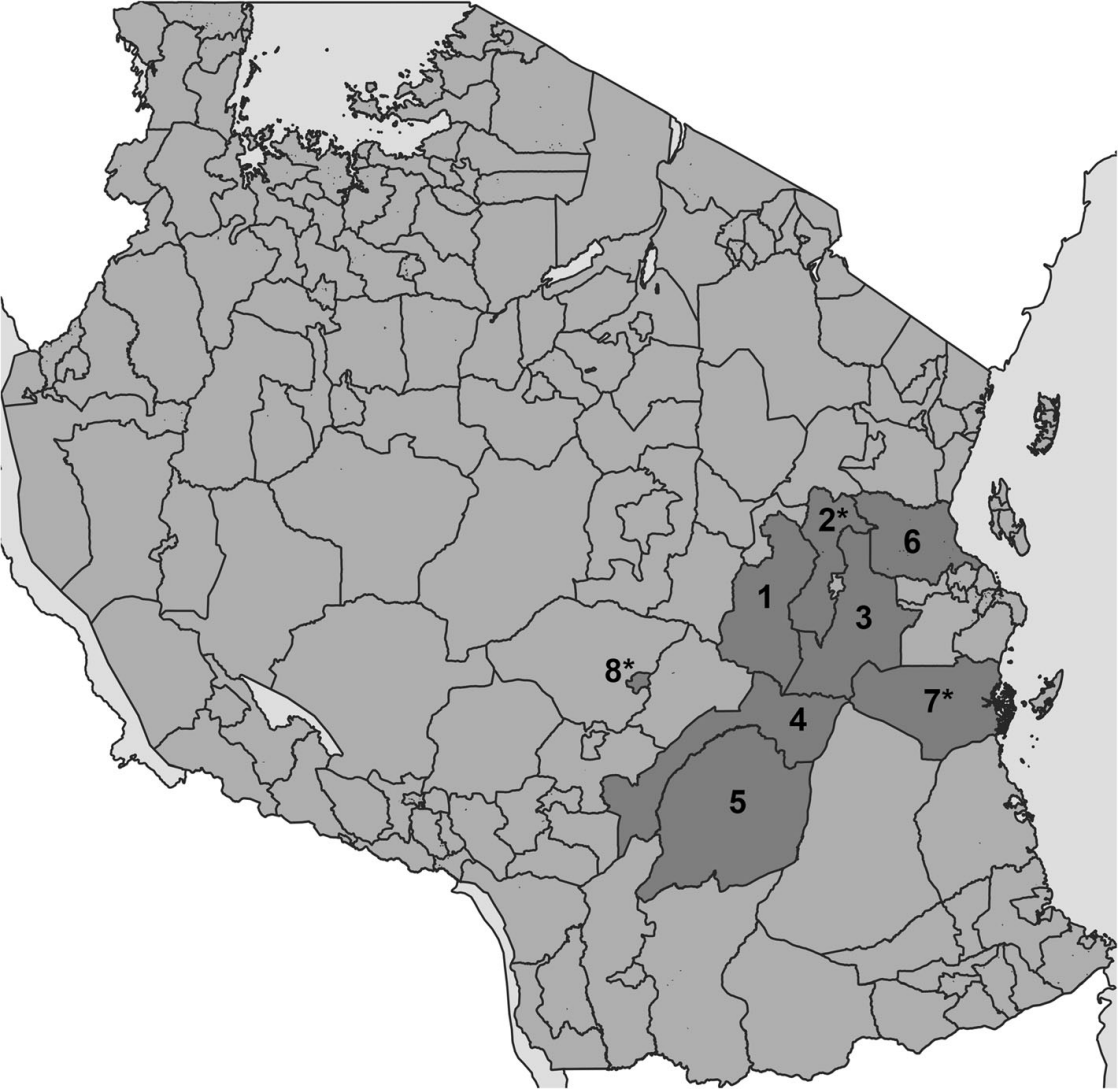
Map of Tanzania with councils where the e-TIQH supportive supervision approach was implemented (status 2012). Morogoro Region: [1] Kilosa DC (later split into Kilosa and Gairo DC), [2] Mvomero DC, [3] Morogoro DC, [4] Kilombero DC, [5] Ulanga DC; Pwani Region: [6] Bagamoyo DC, [7] Rufiji DC; Iringa Region: [8] Iringa MC. Asterisks mark councils selected for qualitative data collection. Map was generated by the authors using QGIS software and shapefiles obtained from the National Bureau of Statistics in Tanzania

**Fig. 3:**
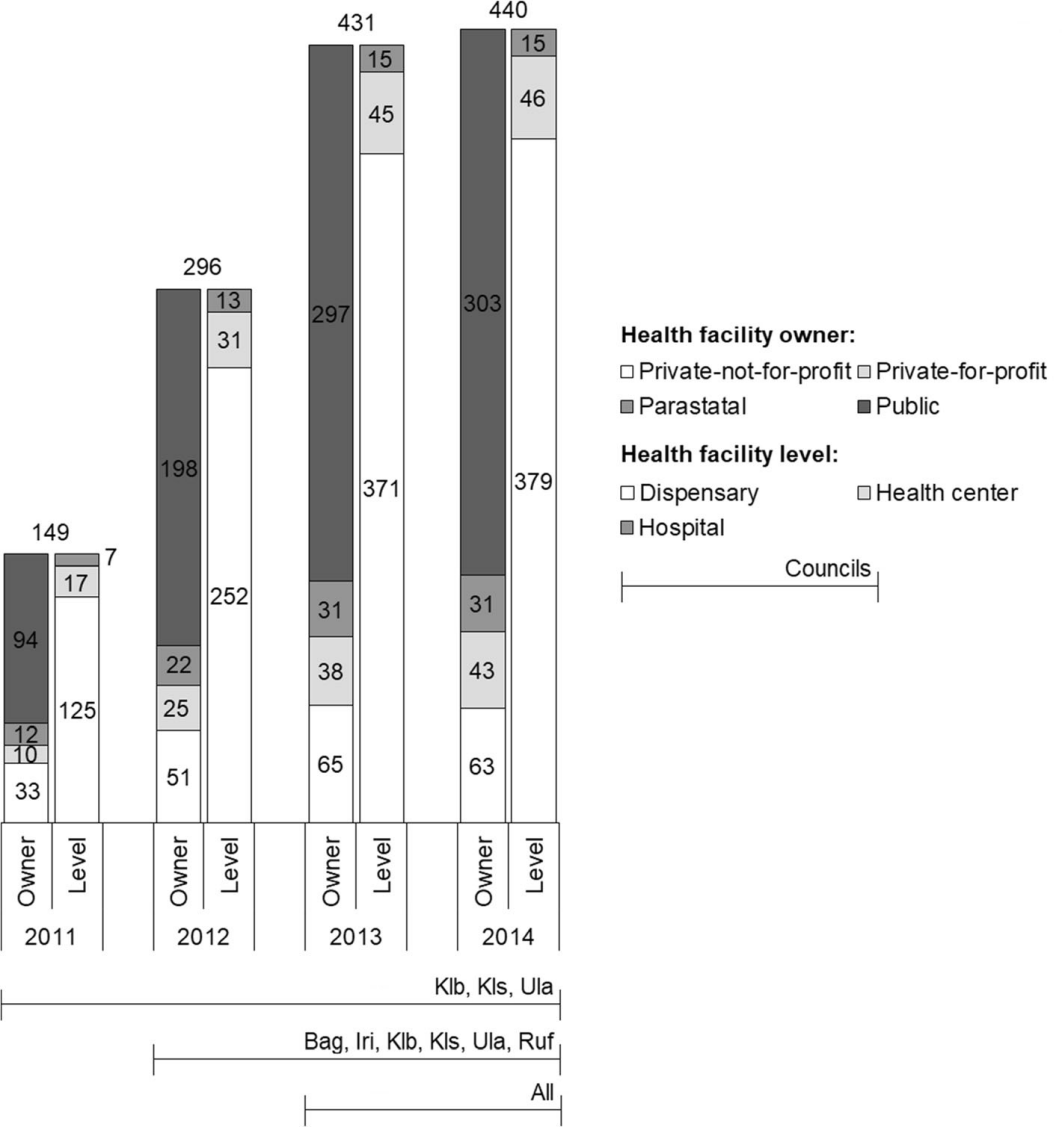
Number of health facilities assessed in each year by health facility owner and level category across selected councils (horizontal lines at bottom). Bag = Bagamoyo DC, Iri = Iringa MC, Klb = Kilombero DC, Kls = Kilosa DC (later split into Kilosa and Gairo DC), Mor = Morogoro DC, Mvo = Mvomero DC, Ruf = Rufiji DC, Ula = Ulanga DC

The new supportive supervision approach, which was developed as part of the “Initiative to Strengthen Affordability and Quality of Healthcare (ISAQH)”, consisted of three stages [56]. In a first step a systematic assessment of quality of primary care was carried out in all health facilities within a given council, using the “electronic Tool to Improve Quality of Healthcare” – in short e-TIQH (Fig. 1). CHMT members formed the core of the assessment team. They were supported by community representatives and healthcare providers from the public and private sector. Assessment supervision was done by ISAQH staff. The assessment methods included checklists, structured interviews and direct clinical observations. Importantly, the assessment concluded with an immediate constructive feedback to the healthcare providers and joint discussions about how to address the identified quality gaps. In a second step, a dissemination meeting was held at council level with all relevant stakeholders to discuss the findings and develop action plans. This provided important inputs for the third step, the annual council health planning and budgeting process. Using the e-TIQH supportive supervision approach data on quality of primary healthcare was electronically gathered between 2011 and 2014 in health facilities in up to eight Tanzanian district and municipal councils (DCs and MCs) (Fig. 2). Table 1 summarizes the characteristics of the councils. Due to a phased introduction of the e-TIQH approach, the number of assessed councils and health facilities varied from 1 year to the other (Fig. 3). Kilombero and Ulanga DC were pilot councils for a paper-based version of the same tool. Bagamoyo, Kilosa and Rufiji DC as well as Iringa MC were selected because they had improved health data systems in place thanks to the Sentinel Panel of Districts [59]. Mvomero and Morogoro DC were included due their proximity within the main region of operation. In total, six quality dimensions containing 183 indicators were consistently assessed over all 4 years: [1] Physical environment and equipment [2]; Job expectations [3]; Professional knowledge, skills and ethics [4]; Management and administration [5]; Staff motivation [6]; Client satisfaction. The dimensions and indicators were developed in an iterative process by the ISAQH staff together with key stakeholders. This process strictly followed existing national treatment, supportive supervision, and other guidelines [56]. Points were given for each indicator met, and percentage scores (of total possible points) were calculated per quality dimension. The score of each quality dimension then equally contributed to the overall health facility score [56].

**Table 1:** Description of councils where the e-TIQH supportive supervision approach was implemented (status 2014)

Region/District	Rural/Urban	Population [58]	Number of health facilities		
			Dispen-saries	Health centres	Hospital
Morogoro Region					
Ulanga DC	Rural	265′203	33	3	2
Kilombero DC	Rural	407′880	52	5	2
Kilosa/Gairo DC	Rural	631′186	69	9	3
Mvomero DC	Rural	312′109	52	8	3
Morogoro DC	Rural	286′248	54	7	0
Iringa Region					
Iringa MC	Urban	151′345	21	4	3
Coast Region					
Bagamoyo DC	Rural	311′740	69	5	1
Rufiji DC	Rural	217′274	62	5	2
Total		**2′582’985**	**412**	**46**	**16**

### Quantitative approach

**Fig. 4:**
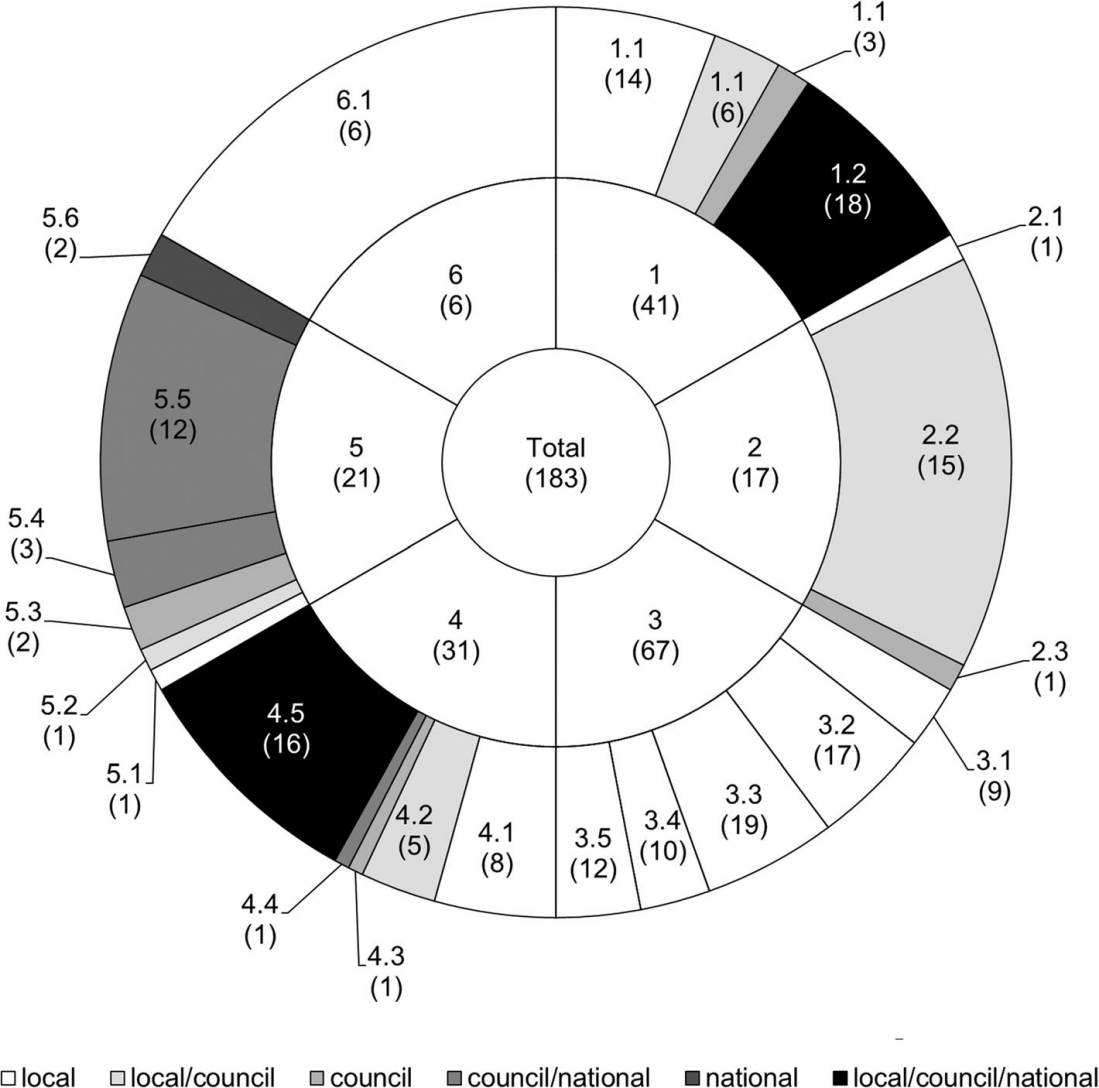
Total number of indicators (inner circle) and the number of indicators per quality dimension (1–6; middle circle) and thematic category by responsible health system level (1.1–6.1; outer circle). Number of indicators is given in brackets. The e-TIQH assessment tool consisted of six quality dimensions contributing equally to the overall score, which is illustrated by the middle circle through equivalent areas of each quality dimension [56]. QD 1 = Physical environment and equipment: QD 1.1 = Physical environment, QD 1.2 = Equipment availability; QD 2 = Job expectations: QD 2.1 = Provider knowledge of services to be provided, QD 2.2 = Guideline and algorithm availability, QD 2.3 = Availability of job description; QD 3 = Professional knowledge, skills and ethics: QD 3.1 = Ethics and Infection Prevention and Control (IPC), QD 3.2 = Integrated Management of Childhood Illnesses (IMCI), QD 3.3 = Maternal health, QD 3.4 = Fever, QD 3.5 = HIV/AIDS and TB; QD 4 = Management and administration: QD 4.1 = Display of public information, suggestion box, meeting conduction, duty roster, referral plans, QD 4.2 = Information, Education and Communication (IEC) material availability and Health Management Information System (HMIS) implementation, QD 4.3 = Routine CHMT supportive supervision visits, QD 4.4 = Staffing level, QD 4.5 = Medicines availability; QD 5 = Staff motivation: QD 5.1 = In-house education, QD 5.2 = Appointment as best worker, QD 5.3 = Letter of appreciation and training follow up, QD 5.4 = Reward payment, house allocation, promotion, QD 5.5 = Training, QD 5.6 = Salary and promotion payment; QD 6 = Client satisfaction

To identify the drivers of quality improvements, indicators of the six quality dimensions were further grouped into thematic categories. For each indicator within these thematic categories we also identified the part of the health system, whose primary responsibility it was to address the indicator (Fig. 4). This categorization was done in consultation with a local medical expert familiar with the assessment procedures. Responsibilities could be shared between more than one level, leading to six groups: indicators that primarily ought to be addressed at local (l), council (c) or national (n) level, or in collaboration at local and council (l/c), council and national (c/n), or all (l/c/n) levels. For public providers, the local level included the staff working at local health facilities and the council and national level the stakeholders acting at council and national level, respectively (e.g. the CHMTs at council level). For private providers, the local level was seen as the staff directly in contact with the client, the council level as the local management level, and the national level as the management at the highest level, e.g. an umbrella institution or owner, which could potentially even be based outside the country. To assess trends over time we calculated the overall score (inner circle, Fig. 4), the six quality dimension scores (middle circle, Fig. 4) and the scores for each thematic category by responsible health system level (outer circle, Fig. 4) for every health facility and year. Afterwards mixed linear regression models were derived for each of these scores. Year was included as a categorical variable (2011, 2012, 2013, 2014) and the variable council was set as a random effect. The following equation presents the random effect regression model of the overall score for health facility i in council j: *HFscore*_*ij*_ = *β*_0_ + *β*_1_*year*2_*ij*_ + *β*_2_*year*3_*ij*_ + *β*_3_*year*4_*ij*_ + *u*_*j*_ + *e*_*ij*_ [1] u_j_ is the random effect for council and e_ij_ the random effect for health facilities within a council (error term). Regression models for the other scores were in-line with the example given in eq. 1. In a predecessor paper, which used the same database, additional categorical variables (health facility level and health facility owner) as well as third and second order interaction terms were included [60]. The latter were stepwise excluded using Wald test, whereby the variable with the highest order and *p*-value was excluded first. Models without any interaction terms performed best. In this paper no additional categorical variables were included in order to ease comparing the models for the different scores. Yet, comparisons between the models presented here and models including all additional categorical variables [60] were done to check for differences in significance of coefficients. Also, we did a sensitivity analysis to compare the random effect model (equation 1) with a fixed effect model (equation 2) using the robust variance estimator. *HFscore*_*ij*_ = *β*_0_ + *β*_1_*year*2 + *β*_2_*year*3 + *β*_3_*year*4 + *β*_4_*council*2 + *β*_5_*council*3 + *β*_6_*council*4 + *β*_7_*council*5 + *β*_8_*council*6 + *β*_9_*council*7 + *β*_10_*council*8 + *e*_*ij*_ [2] To do so, the relative difference between a given coefficient in the random effect model and the same coefficient in the fixed effect model was calculated for coefficients with a *p*-value lower than 0.05 in at least one of the models. This was done for each coefficient of all regression models.

### Qualitative approach

We used qualitative data to support and supplement quantitative findings and to investigate whether and how the e-TIQH supportive supervision approach contributed to changes in quality of care. Qualitative data therefore intended to identify areas in which quantitatively captured quality trends could at least to some extent be attributed to the e-TIQH approach. We aimed to strengthen these findings through triangulation of methods and consistency across councils. Alternative methods to assess attribution of quality improvements to the e-TIQH approach were not feasible because this research was analyzing an implementation project. The main part of the qualitative data consisted of in-depth interviews, whereas observational data and informal personal communication recorded in a field notebook as well as materials collected during the field work complemented the data set. A total of 24 in-depth interviews were conducted in three out of eight intervention councils (Fig. 2) because of the limited resources available for this study. However, this did not hamper the quality of the qualitative study because saturation of information was reached prior to the completion of all 24 interviews. Since we aimed to understand reasons for changes in quality of care, the councils with the biggest yearly changes in overall quality (as measured by the e-TIQH assessments) were selected. Sampling coincidentally resulted in the selection of three councils, which were very different in terms of their characteristics (Table 2). This ensured representativeness and facilitated drawing conclusions for a wide range of contexts within Tanzania. Sampling of interview partners was done purposefully. At council level two CHMT members (including co-opted members) were interviewed as representatives of the public sector. Additionally, two members of the Council Health Service Board (CHSB), which is the governance body responsible for adequate service delivery and CHMT oversight at council level, were selected to represent the non-public sector [64]. Within the rural councils we selected one public health center and one well and one less well performing public dispensary in terms of quality of care (as measured by the e-TIQH assessments). For the urban council we chose one well and one less well performing dispensary (as measured by the e-TIQH assessments) from the public and the private sector each. Interviews were done with the facility in-charge, and in health centers also with the person responsible for quality improvements (Table 3). Table 4 summarizes some of the demographic characteristics of the respondents. To ensure confidentiality, no further information about the respondents could be given here. For being considered as a respondent, the respondent had to be in the respective position for at least part of the time period in which the e-TIQH approach had been implemented or have comparable experience, based on the interviewers’ judgement. Written informed consent was obtained from all respondents. Interviews were conducted in the first quarter of 2016. They were done in Swahili and led by a Swahili speaking female Swiss (SR). She was backed up by a male native Tanzanian of middle age (IM). Interviews were guided by the main question relating to whether and how quality of care changed over time and why. It was ensured that respondents clearly refer to a time period in order to assign an event to the timespan before, after or in which the e-TIQH approach had been implemented. It was also probed for specific areas of potential improvements. These areas were based on the health system building blocks (service delivery, health workforce, information/research, healthcare financing, medical product/technology, leadership/governance), as defined by the World Health Organization’s health system framework [65]. The health system building blocks were chosen to allow capturing improvements across the whole system, not necessarily only areas included in the e-TIQH assessment tool. The e-TIQH quality dimensions and their thematic categories presented above in Fig. 4 were used as sub-areas within the corresponding building block [65]. Importantly, it was never directly asked if the e-TIQH supportive supervision approach led to certain changes. All interviews were tape-recorded and transcribed by two native Tanzanian research assistants but not translated into English. The transcripts were managed and coded with MAXQDA software. Data were analyzed using the framework method described by Gale et al. [66], which uses a structured matrix output to systematically reduce and analyze qualitative data. Coding was primarily done deductively. We used the six health system building blocks as themes. Categories were developed in-line with the e-TIQH thematic categories with space for induction, which allowed creating categories not covered by the e-TIQH assessment tool. Findings were compared for similarities and differences within and between respondent groups. To do so, we took into account the respondent’s gender, age, time in the respective position, position and their working environment (council, health system level and ownership of health facility). Citations used in the text were translated by SR into English and proofread by IM.

**Table 2:** Description of councils selected for the qualitative study

Characteristics	Rufiji DC	Mvomero DC	Iringa MC
Region	Pwani	Morogoro	Iringa
Classification	Rural	Rural	Urban
Population size [58]	217′274	312′109	151′345
Area (km^b^)^a^	13′339	7′325	162
Number of operating health facilities [61]^b^	78	69	33
Accessibility	Several hard-to-reach areas, including the Rufiji river delta	Some hard-to-reach areas	No hard-to-reach areas
Existence of pay for performance (P4P) schemes (20)^c^	Pilot council for donor funded P4P scheme since 2011 with focus on maternal, newborn and child health services [62]	Partially implemented locally funded P4P scheme between 2009 and 2011 with focus on maternal, newborn and child health services [63]	No P4P experience

^a^Source: Comprehensive Council Health Plans of participating councils collected by SR and IM ^b^Status October 2016 ^c^Result-based financing scheme whereby financial incentives, which are tied to the achievement of service coverage and/or quality improvements, are provided to the healthcare provider

**Table 3:** Number of in-depth interviews done in the three study councils (Mvomero DC/ Rufiji DC/Iringa MC)

Position	Administrative level	Sector	
		Public	Non-public
CHMT (co-opted) member	Council	2/2/2	
CHSB member	Council		2/2/2
Health center in-charge	Health center	1/1/0	
Quality improvement person	Health center	1/1/0	
Dispensary in-charge	Dispensary	2/2/2	0/0/2
Total		16	8

**Table 4:** Demographic characteristics of the respondents

	CHMT member (*n* = 6)	CHSB member (*n* = 6)	Health center in-charge (*n* = 2)	Quality improvement person (*n* = 2)	Dispensary in-charge (*n* = 8)
In position since [years]					
< 2.5 (*n* = 5)	1	4	0	0	0
2.5–4.5 (*n* = 8)	2	0	1	1	4
5–7 (*n* = 6)	1	2	0	1	2
> 7 (*n* = 5)	2	0	1	0	2
Gender					
male (*n* = 15)	4	5	2	0	4
female (*n* = 9)	2	1	0	2	4
Age [years]					
< 40 (*n* = 6)	3	0	0	0	3
40–49 (*n* = 3)	0	1	1	0	1
50–59 (*n* = 11)	3	2	1	2	3
> 59 (*n* = 4)	0	3	0	0	1

## Results

### Trends in quality of care as measured by the e-TIQH assessment tool

**Fig. 5:**
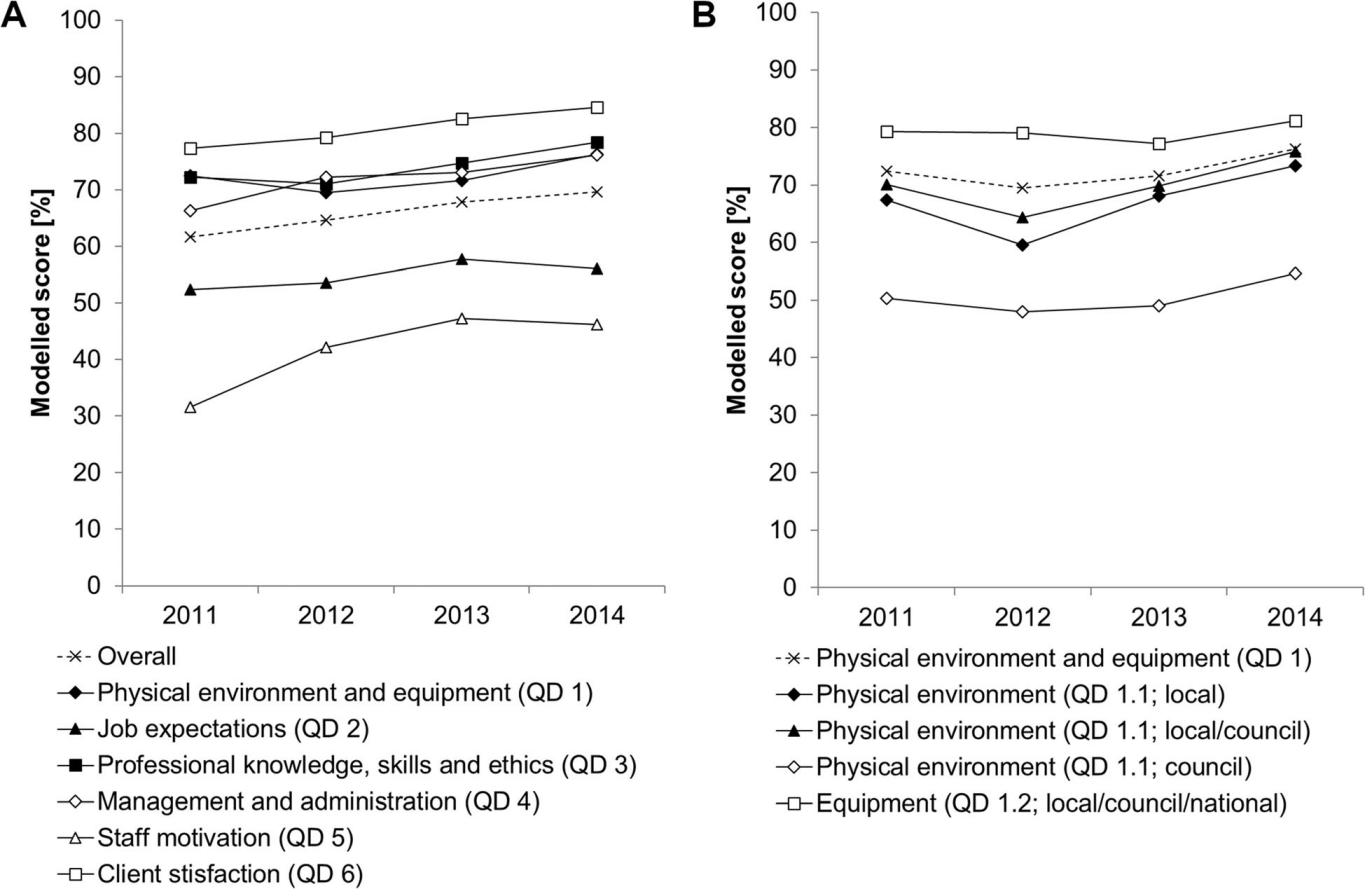
Time trends for performance by quality dimensions (**a**) and by thematic categories and responsible health system level of quality dimension 1 (**b**)

Differences in average quality dimension (QD) and thematic category scores, expressed as percentages of maximum achievable scores for the years 2012 to 2014 are given in Table 5. These linear regression coefficients indicate how the scores of each quality dimension and thematic category changed compared to the year 2011. The year 2011 percentage score is given by the constant. The sensitivity analysis showed that most coefficients with a *p*-value below 0.05 in the random or fixed effect model were similar to the same coefficient in the other model. The coefficients which differed by more than 10% of their means are referred to with a hashtag in Table 5. Thus, there was no major difference between the random and fixed effect model using the robust variance estimator. For illustrative purposes, time trends are also shown graphically in Fig. 5 for performance of each quality dimension as well as thematic category and responsible health system level for quality dimension 1. For quality dimension 1, which summarized performance in physical environment and equipment, the indicator groups that drove the overall improvement of 3.9 percentage point the most belonged to the category physical environment. These indicators could be addressed either at local level (6.0 percentage point increase) or in collaboration by the local and council level (5.7 percentage point increase). In quality dimension 2, which assessed job expectation, the availability of guidelines and algorithms significantly increased between 2011 and 2014 (4.9 percentage point increase), while trends in the availability of job descriptions went in the opposite direction during the same time period (11.3 percentage point decrease). This resulted in an insignificant overall improvement of 3.7 percentage points. Results of quality dimension 3 revealed that improvements in performance of clinical consultations between 2011 and 2014 varied across categories. They were significant for all types of consultations except when assessing fever cases in patients above 5 years of age. Significant improvements ranged from 4.1 percentage points for maternal health consultations to 20.1 percentage points for HIV/AIDS and TB patients. Quality dimension 4, which represented a broad spectrum of management and administration issues, showed significant positive trends between 2011 and 2014 for the categories capturing medicine availability (8.1 percentage point increase) and things that could be addressed at local level or in collaboration by the local and council level (QD 4.1 11.6 and 4.2 16.9 percentage point increase). In quality dimension 5, which incorporated different types of incentives to boost staff motivation, all categories changed significantly over time. Percentage point increases ranged from 7.5 for appointing best worker and 21.1 for timeliness of salary and promotion payment. Lastly, client satisfaction as measured in quality dimension 6 also indicated a positive trend from 2011 to 2014 with 7.3 percentage point increase.

**Table 5:** Differences in average quality dimension (QD) and thematic category scores, expressed as percentages of maximum achievable scores, according to year, while the variable council was set as a random effect

Performance by quality dimension (QD)								
	Overall	QD 1	QD 2	QD 3	QD 4	QD 5	QD 6	
2012	3.0**	−2.9*	1.3	− 1.2	5.9***	10.6***	1.9	
2013	6.2***	− 0.9	5.4*	2.4	6.7***	15.8***	5.2***	
2014	8.0***	3.9**	3.7^(58)°^	6.2***	9.9***	14.6***	7.3***	
Constant	61.6***	72.5***	52.4***	72.3***	66.3***	31.5***	77.4***	
QD1: Physical environment and equipment by thematic category and responsible health system level								
	QD 1.1, (l)	QD 1.1, (l/c)	QD 1.1, (c)	QD 1.2, (l/c/n)				
2012	−7.9**	−5.7*	−2.2	− 0.2				
2013	0.7	− 0.2	− 1.3	− 2.1				
2014	6.0**	5.7*	4.4	1.9				
Constant	67.4***	70.0***	50.3***	79.3***				
QD 2: Job expectations by thematic category and responsible health system level								
	QD2.1, (l)	QD 2.2, (l/c)	QD2.3, (c)					
2012	−2.3	0.2	15.7***					
2013	1.1	5.4*	7.1					
2014	0.4	4.9*	−11.3**					
Constant	97.8***	49.1***	54.6***^#i^					
QD 3: Professional knowledge, skills and ethics by thematic category and responsible health system level								
	QD 3.1, (l)	QD 3.2, (l)	QD 3.3, (l)	QD 3.4, (l)	QD 3.5, (l)			
2012	−7.0***	−4.3	4.3*	−4.8	15.6***			
2013	−3.5*	4.4	2.2	2.4	16.0***			
2014	4.4**	7.3**	4.1*	2.2	20.1***			
Constant	78.2***	67.0***	81.7***	66.2***^#ii^	76.3***			
QD 4: Management and administration by thematic category and responsible health system level								
	QD 4.1, (l)	QD 4.2, (l/c)	QD 4.3, (c)	QD 4.4, (c/n)	QD 4.5, (l/c/n)			
2012	−0.6	14.1***	−9.4*	−1.6	8.2***			
2013	3.5	15.1***	2.8	−3.6	7.1***			
2014	11.6***	16.9***	5.5	6.1	8.1***			
Constant	53.8***^#iii^	55.2***	84.7***	27.9***^#iv^	75.7***			
QD 5: Staff motivation by thematic category and responsible health system level								
	QD 5.1, (l)	QD 5.2, (l/c)	QD 5.3, (c)	QD 5.4, (c/n)	QD 5.5, (c/n)	QD 5.6, (n)		
2012	7.2	2.4	8.7**	13.1***	10.0***	12.3***		
2013	10.9**	4.2^#v^	18.6***	12.6***	16.9***	14.4***		
2014	15.7***	7.5**	18.9***	9.2**	15.1***	21.1***		
Constant	59.8***	7.4**^#vi^	38.2***	35.3***^#vii^	23.5***	67.1***		

Asterisks refer to *p*-values indicating the significance of a coefficient *p < 0.05, **p < 0.01, ***p < 0.001 °Coefficient that would have been significant in a model including additional categorical variables (health facility level and health facility owner) but was not in the model presented here [60]. ^#^Coefficients with a *p*-value below 0.05 in the random or fixed effect model and which differed by more than 10% of their means: (i) random: 54.6*** (*p* = 0.000), fixed: 36.0 (*p* = 0.000); (ii) random: 66.2*** (*p* = 0.000), fixed: 73.3*** (*p* = 0.000); (iii) random: 53.8*** (*p* = 0.000), fixed: 45.1*** (*p* = 0.000); (iv) random: 27.9*** (*p* = 0.000), fixed: 23.8*** (*p* = 0.000); (v) random: 4.2 (*p* = 0.102), fixed: 4.8* (*p* = 0.038)); (vi) random: 7.4*** (*p* = 0.006), fixed: 2.2 (*p* = 0.399); (vii) random: 35.3*** (*p* = 0.000), fixed: 31.7*** (*p* = 0.000) There was a large fraction of unexplained variance attributed to the random effect for all models, meaning that scores were strongly correlated within councils (data not shown). Responsible health system levels are given in brackets for easier reference: l = local, c = council; *n* = national QD 1 = Physical environment and equipment: QD 1.1 = Physical environment, QD 1.2 = Equipment availability; QD 2 = Job expectations: QD 2.1 = Provider knowledge of services to be provided, QD 2.2 = Guideline and algorithm availability, QD 2.3 = Availability of job description; QD 3 = Professional knowledge, skills and ethics: QD 3.1 = Ethics and Infection Prevention and Control (IPC), QD 3.2 = Integrated Management of Childhood Illnesses (IMCI), QD 3.3 = Maternal health, QD 3.4 = Fever, QD 3.5 = HIV/AIDS and TB; QD 4 = Management and administration: QD 4.1 = Display of public information, suggestion box, meeting conduction, duty roster, referral plans, QD 4.2 = Information, Education and Communication (IEC) material availability and Health Management Information System (HMIS) implementation, QD 4.3 = Routine CHMT supportive supervision visits, QD 4.4 = Staffing level, QD 4.5 = Medicines availability; QD 5 = Staff motivation: QD 5.1 = In-house education, QD 5.2 = Appointment as best worker, QD 5.3 = Letter of appreciation and training follow up, QD 5.4 = Reward payment, house allocation, promotion, QD 5.5 = Training, QD 5.6 = Salary and promotion payment; QD 6 = Client satisfaction

### Contribution of the e-TIQH supportive supervision approach to improvements in quality of care

A total of 22 out of the 24 respondents directly experienced the e-TIQH approach, either as an assessor, as the person being assessed or during the council dissemination meeting. The subsequent analysis is therefore restricted to these 22 people, because only they could potentially attribute any changes in quality of care to the e-TIQH approach. The following section is structured according to the e-TIQH quality dimensions and focuses on thematic categories in which the e-TIQH supportive supervision approach contributed to improvements. Physical environment and equipment – quality dimension 1 Regarding physical environment and equipment, almost everyone (21 of the 22 included in the analysis) noticed improvements in physical environment. The issues that had been addressed were mostly within the responsibility of the local level, either solely (21 of the 21 above mentioned) or in collaboration with the council (16/21), and rather less frequent in the hands of the councils (11/21). This was in-line with quantitative findings showing significant improvements at local level or in collaboration by the local and council level, but non-significant improvements at council level (Table 5, QD 1.1). Importantly, a considerable number of respondents attributed improvements in physical environment to a large extent to the e-TIQH approach’s capability to induce improvement measures (19/21 at local, 11/16 at local/council and 6/11 at council level). A CHMT member summarized this as follows: “…the issue of IPC [Infection Prevention and Control]… was very unsatisfactory…we didn’t even have dustbins to dump the waste and also… we didn’t do the segregation of it. And when we passed by [at the health facilities] the first time, they put it [the waste] without looking at the color [of the bins]... If you pass by now waste segregation is done and waste is put according to the type of waste. “Routine CHMT supportive supervision, which complements the e-TIQH approach, was also brought up for having contributed to positive changes in physical environment (6/21 at local, 4/16 at local/council and 4/11 at council level). Other than the CHMT supportive supervisions, P4P schemes (Table 2), the CHSB and other stakeholders were stated for having influenced improvements in physical environment. In the case of P4P schemes this was mainly for gaps that had to be addressed at local level or in collaboration by the local and council level. In contrary, other stakeholders were more involved in things that were partially or fully in the responsibility of the council. Improvements in the availability of appropriate equipment were hardly brought up (6/22), which was in agreement with findings in Table 5 (QD 1.2). If so, they were attributed to a mix of interventions, including the e-TIQH approach (4/6), the availability of CHF money (2/6), non-governmental support (1/6) and P4P schemes (1/6). Job expectations – quality dimension 2 For job expectations, a considerable number of respondents (13/22) reported improvements in guideline and algorithm availability, which was concurrent with the quantitative trend (Table 5, QD 2.2). It was explained that the e-TIQH approach (10/13) and/or routine CHMT supportive supervision (4/13) identified the lack of latest guidelines and algorithms, upon which healthcare providers and CHMTs started initiatives to increase their availability. This was illustrated by a former facility in-charge as follows: “…we practiced [the procedure] assuming we understand… often we didn’t see the importance of having these guidelines, but these guidelines are good. Sometimes you realize… new ones have arrived with changes. It’s easy to open and read them. Thus, its [the e-TIQH approach’s] job was to remind us that… it’s important to have these guidelines. …and because we were with the CHMT it was easy… He/She [the e-TIQH assessor] told you this guideline you can find there [in the office of the CHMT]… this guideline we don’t have, [but] after some days come and look, you will find them [there]. Therefore, it was easy for us to do follow up.” Few respondents additionally elaborated that during the e-TIQH supportive supervision approach (3/13) and/or trainings (2/13) it was emphasized that provided guidelines need to be at work and not at home. Lack of guidelines at council level was seen as the main obstacle for further improvements in this area. Improvements in the availability of job descriptions were barely reported (2/22), which was consistent with the negative trend seen in Table 5 (QD 2.3).Professional knowledge, skills and ethics – quality dimension 3 Changes in performance during clinical consultations as measured by direct observation could have been influenced by several factors, including guideline and algorithm availability, trainings carried out by various stakeholders, as well as supervision visits and in-house education sessions (Table 5, QD 3). These factors therefore have to be accounted for when looking into reasons for improvements of performance during clinical consultations. Half of the respondents (11/22) said that the behavior during consultation, in particular friendliness, provider attitude and language used, improved. A majority of the respondents (8/11) elaborated that it was the e-TIQH approach’s particular emphasis on consultation ethics, which triggered these improvements. Together with the e-TIQH approach, routine CHMT supportive supervision on its own (2/8) or in combination with increased availability of guidelines and in-house training (1/8) were raised. There were also some (3/11), who mainly attributed changes in ethics to stronger community oversight (2/3) and/or trainings conducted (2/3). Apart from consultation ethics, several respondents (8/22) reported improvements in compliance with IPC procedures during consultation. All of them (8/8) said that the direct observations and subsequent feedback of the e-TIQH approach, which was seen as on-job training, contributed to a great extent to these changes. Lastly, a substantial number of respondents (15/22) asserted that treatment guidelines were more closely followed than previously. For example, a facility in-charge said: “… the feedback helped to change us regarding [our] performance because sometimes we forget these steps [of the guidelines], we skip them…we work as we got used to, but… when they [the e-TIQH assessors] did this supervision and the way they did it…it changed us a lot.” All of them (15/15) acknowledged that the e-TIQH observational approach contributed to these changes. Some also added in-house training (1/15), trainings conducted by other stakeholders (2/15) and routine CHMT supportive supervision (1/15) were leading to improvements. Interestingly, in one council, routine CHMT supportive supervision was subsequently improved by using the same observational approach. Management and administration – quality dimension 4 With respect to management and administration some respondents reported positive changes in the category capturing things that could be addressed at local level (7/22). They uniquely said that these were triggered by the e-TIQH intervention (7/7). However, all other significant improvements in Table 5 were barely due to the e-TIQH approach, but rather because of other interventions. For example, respondents (7/22) acknowledged considerable improvements in respect to the Health Management Information System (HMIS) reporting. Though, none of them mentioned the e-TIQH approach for having initiated these changes. They rather stated tight follow up from council level (4/7), better health facility internal organization (2/7), increased number of staff (2/7) and improved HMIS system (2/7) as reasons for better HMIS reporting. Likewise, better medicine supply was mainly raised (15/22) in conjunction with improved supply chain management (7/15) and availability of additional health financing mechanisms (8/15), rather than with the e-TIQH approach (2/15). Staff motivation – quality dimension 5 A considerable number of respondents stated that due to the e-TIQH approach discussions around required measures to improve staff motivation through benefits and rewards were stimulated or reinitiated (8/22). In this regard a member of the CHSB said: “It’s not that [the] e-TIQH [approach] only showed [us the problems of staff motivation], it stimulated us further [and] made it clearer. The problem however was there since long and people knew it. But… it wasn’t an area about which people were complaining… They [the CHMT] may go to facilities and start talking about other things, but staff benefits were not spoken about… but [the] e-TIQH [approach] goes as far as asking about staff benefits, you see? The problem was there, but it was not spoken about because it wasn’t seen as [the CHMT’s] responsibility to ask, but [the] e-TIQH [approach] sees it as its responsibility to ask the personnel. Is he/she satisfied with the work he/she is doing? Is he/she feeling appreciated? Does he/she get the salary on time?” Some improvements were subsequently implemented, whereas the respondents in particular highlighted non-financial benefits. This suggested that the e-TIQH supportive supervision approach potentially contributed to some of the improvements presented in Table 5, QD 5.2, 5.3, and 5.4. In two councils P4P schemes were mentioned (10/22) in the context of reward payments. However, despite the positive changes shown in Table 5 complains with regard to benefits and rewards remained high, especially regarding financial employment benefits. According to the respondents the main problems were insufficient and delayed allocation of money from the national level to the councils, and lack of knowledge about administrative procedures at local and council level. For the category “training”, some respondents confirmed the positive trends seen in Table 5 (3/22) while others stated the opposite (3/22), but the e-TIQH approach was hardly brought up in this context. Finally, improvements in timeliness of wage and promotion payments could almost solely be attributed to a revised payment process implemented by the national government as unanimously reported by respondents (Table 5, QD 5.6).

## Discussion

Using a mixed methods approach, we aimed to identify drivers of quality improvements and examine whether the e-TIQH supportive supervision approach was able to contribute to these improvements. The results presented showed that the qualitative and quantitative findings were overlapping and strongly consistent. This strengthened the here identified trends and drivers of quality improvements. It also supported preliminary findings and quality trends documented by Mboya et al. [56] and confirmed what was demonstrated regarding the tool’s appropriateness to accurately assess quality of primary healthcare [60].

### Contribution of the e-TIQH supportive supervision approach to quality improvements

Qualitative data identified areas in which the e-TIQH supportive supervision approach contributed to improvements. Advances in physical environment that could be implemented at local level with or without the help of the council could largely be attributed to the e-TIQH approach. The e-TIQH approach also helped to address issues in physical environment, where the responsibility lied with the councils. The approach could therefore reduce some of the problems around insufficient health infrastructure, poor healthcare waste disposal and low hygiene and sanitation standards [16, 17]. Apart from physical environment, availability of guidelines and algorithms was another category in which improvements were seen in connection with the e-TIQH intervention. Additionally, although acknowledging the likely contribution of trainings conducted by other stakeholders, the direct clinical observations and subsequent feedback of the e-TIQH approach made an important contribution to improved performance during clinical consultations as hypothesized previously [56]. This suggested that the e-TIQH supportive supervision approach not only led to structural changes, but also improved processes. It also demonstrated that measuring process quality by means of observations followed by appropriate immediate feedback positively affected provider practice. In our study, direct observations were highly beneficial for healthcare providers and seen as on-job training, despite the criticism of observations as a process measure [67]. This was in line with what was found and recommended by others in particular in respect to onsite training follow-up visits [27, 31, 33, 52, 68–71]. Consequently, the know-do gap and problems with inadequate provider adherence to professional and ethical conduct could be decreased [16, 17]. For management and administration issues, the e-TIQH approach considerably contributed to the improvements in areas that ought to be addressed at local level. An additional area, which was likely to be positively affected by the e-TIQH approach, was the improved provision of non-financial staff benefits. There, the approach had a crucial role in providing solid evidence about the sensitive topic of staff benefits, and therewith made it possible to officially discuss the issue. This may have reduced the problem of low health worker motivation [16, 17]. Overall, we conclude that the e-TIQH supportive supervision approach led to improvements that could be solved at local level, either solely by the healthcare provider or in collaboration with the council. The immediate, supportive feedback followed by solution-oriented discussions with those who were able to address the identified problems was key to the approach. The approach therewith managed to address the lack of feedback on performance upon health facility assessments, as raised previously [14]. This fostered ownership of quality improvement measures at facility level [16, 17]. Additionally, if the collected data was used appropriately, the e-TIQH approach could also inform improvement measures that needed to be taken at council level. It therefore considerably reduced the problem of poor feedback on quality developments at council level [16, 17]. Finally, although the e-TIQH approach mainly contributed to improvements that required no or little financial means, it facilitated the process of priority setting at local and council level in the light of limited resources.

### Contribution of other interventions to quality improvements

In all the above mentioned improvement processes, healthcare providers and CHMTs were crucial in the implementation of improvement measures. This was because of the participatory e-TIQH approach with strong involvement of local and council stakeholders. Likewise, adoption of the routine CHMT supportive supervision procedures upon exposure to the e-TIQH approach played a key role. This however also meant that contributions made by CHMT supportive supervision could not be clearly distinguished from the direct contributions of the e-TIQH supportive supervision approach as they were complementary. Additionally, revenue collection at health facility level, e.g. through health financing mechanisms and P4P schemes, enabled providers to take and finance actions. This was important for compensating the lack of enough financial means from council and national level to implement the improvement measures at health facility level. It was also in-line with previous findings regarding the use of such kind of revenues [72–75]. Observational data further suggested that a self-assessment approach focusing on physical environment (as described by Kamiya et al. [76]) might have as well led to positive changes in physical environment at local level. Improvements in equipment and medicine availability were hardly influenced by the e-TIQH approach as this had largely to be addressed at council and national level and with substantial financial resources. For the same reasons there was no clear contributions of the e-TIQH approach to increased numbers of trainings or improvements in management and administration above the local level. Improvements that ought to be addressed at council level also often required considerable financial resources. Here, other stakeholders, especially non-governmental organizations, seemed to have contributed to these improvements. Lastly, there was no contribution of the e-TIQH approach regarding improved timeliness of salary and promotion payments, or positive changes in staff motivation, that needed a substantial amount of money from national level for its implementation.

### Limitations of the study

It is recognized that well-trained assessors familiar with the context are key for the validity and precision of the assessment and crucial for constructive feedback, an important base for subsequent improvements. In the case of direct observations, a Hawthorne effect could not be excluded [77–79]. We further acknowledge that the presented regression models could have been improved by including additional variables and potentially significant interaction terms. However, comparing the models presented here and models including all additional variables [60] did not lead to a difference in significance of coefficients. Additionally, although conclusions presented here were supported by the triangulation of methods, we recognize that causality cannot conclusively be claimed. Unknown factors might have also contributed to the observed results. We also could not fully exclude that the improvements seen were driven by the choice of the indicators included in the e-TIQH tool, which might have led to overestimation of real changes. The respondents were aware that the interviewers knew the team who facilitated the implementation of the e-TIQH supportive supervision approach. This could have potentially led to statements overestimating the contribution of the e-TIQH approach. Moreover, it was not part of the analysis presented here to investigate improvements in quality of care which were not quantitatively captured through e-TIQH assessments. This included contributions of other stakeholders, and additional benefits of the overall e-TIQH supportive supervision approach, like increased staff motivation owing to appropriate feedback given at health facility. The latter will be discussed in a forthcoming paper, which aims to compare the e-TIQH approach with routine CHMT supportive supervision as it is currently implemented [57]. It was also beyond the scope of this analysis to examine the effects of the e-TIQH-linked quality improvements on changes in health outcomes. Hence, the proof that improved processes lead to improved outcomes could be subject of further research, for example through connecting community health data with health facility data.

## Conclusions

The results clearly demonstrated that the e-TIQH supportive supervision approach not only served to assess quality of primary healthcare, but also to address quality issues that laid within the responsibility of the councils or the health facilities. Hence, the e-TIQH approach was able to improve and maintain crucial primary healthcare quality standards across different health facility level and owner categories in various contexts. It also managed to address several major quality issues outlined in the National Health and Social Welfare Quality Improvement Strategic Plan [17]. To the best of our knowledge this is currently the only approach to directly strengthen routine CHMT supportive supervision in Tanzania that has demonstrated such direct impact on general quality of primary care. The e-TIQH approach therefore presents a powerful tool to support, guide and drive quality improvement measures within councils. It can thus be considered a suitable option to make routine supportive supervision more effective and adequate.

## Data Availability

The quantitative dataset used and analyzed during the current study are available from the corresponding author on reasonable request. The qualitative dataset generated and analyzed during the current study are not publicly available as individual privacy of the respondents could be compromised but are available from the corresponding author on reasonable request.

## Abbreviations

CHMT: Council Health Management Team
CHSB: Council Health Service Board
DC: District Council
e-TIQH: electronic Tool to Improve Quality of Healthcare
HMIS: Health Management Information System
HSSP: Health Sector Strategic Plan
IEC: Information, Education and Communication
IMCI: Integrated Management of Childhood Illness
IPC: Infection Prevention and Control
MC: Municipal Council
NIMR: National Institute for Medical Research
P4P: Pay-for-Performance
QD: Quality dimension
UHC: Universal Health Coverage
